# Fucoidan inhibits lymphangiogenesis by downregulating the expression of VEGFR3 and PROX1 in human lymphatic endothelial cells

**DOI:** 10.18632/oncotarget.9443

**Published:** 2016-05-18

**Authors:** Yazong Yang, Zixiang Gao, Yanhong Ma, Hongming Teng, Zundong Liu, Hengyun Wei, Yanbing Lu, Xiaofang Cheng, Lin Hou, Xiangyang Zou

**Affiliations:** ^1^ Department of Biotechnology, Dalian Medical University, Dalian, 116044, China; ^2^ College of Life Sciences, Liaoning Normal University, Dalian, 116081, China

**Keywords:** fucoidan, lymphangiogenesis, lymphatic metastasis, lymphatic endothelial cells, PROX1

## Abstract

Lymphangiogenesis is one of the promoters of tumor lymphatic metastasis. Fucoidan which is a fucose-enriched sulfated polysaccharide has effect on various pharmacological activities including anti-metastasis activity. However, the inhibitory effect of fucoidan on lymphangiogenesis remains unclear. Here, fucoidan extracted from *U. pinnatifida* sporophylls suppressed HLECs proliferation, migration and tube-like structure formation, and had inhibitory effect of tumor-induced lymphangiogenesis *in vitro*. Additionally, we found that fucoidan had a dose-dependent depressive effect on the expressions of PROX1, vascular endothelial growth factor receptor 3 (VEGFR3), NF-κB, phospho-PI3K and phospho-Akt in HLECs. Moreover, anti-lymphangiogenesis effect of fucoidan was assessed by using mouse tumor model. In summary, fucoidan inhibit tumor lymphangiogenesis and lymphatic metastasis by suppressing the NF-κB/PI3K/Akt signaling pathway through reduced levels of PROX1 and VEGFR3.

## INTRODUCTION

Lymphangiogenesis involves in certain physiological process and pathological phenomena such as chronic inflammation and tumor metastasis. Metastasis of a malignant tumor is a critical characteristic of aggressive cancer and the cause of the majority of cancer deaths. Lymphatic metastasis is a main route for many types of malignant cells to disseminate to distant organs. Tumor cells invade the pre-existing lymphatic vessels of the peripheral region and migrate to distant lymphatic nodes [1, 2], and tumor cell metastasis and adverse outcomes in cancer patients are intimately related [3]. After cells invade the lymphatic system, lymphangiogenesis is induced via the production of lymphangiogenic factors and various mediators. The basic process of lymphangiogenesis includes proliferation and metastasis of lymphatic endothelial cells (LECs) and the formation of lymphatic vessels [4]. Prospero homeobox protein 1 (PROX1) and vascular endothelial growth factor receptor 3 (VEGFR3) are the main regulators of lymphangiogenesis [5, 6]. PROX1, which is a homeobox transcription factor, plays a fundamental role in the differentiation and maintenance of the lymphatic system and upregulates VEGFR3 expression during embryogenesis [7]. VEGFR3 is a typical transmembrane protein and belongs to a family of tyrosine protein kinase receptors. Activated VEGFR3 and its ligands, vascular endothelial growth factor C and D (VEGF-C and D, respectively), specifically stimulate the proliferation and migration of LECs, and promote lymphangiogenesis [8]. Blocking VEGFR3 signaling transduction can inhibit tumor lymphangiogenesis and metastasis [9]. Furthermore, the NF-κB/PI3K/Akt signaling pathway, which is involved in cell proliferation, migration, adhesion and extracellular matrix degradation, is one classic signaling pathway involved in tumorigenesis and metastasis. It has been reported that the key component, NF-κB directly or indirectly stimulate lymphangiogenesis during inflammation [10]. Inhibition of the NF-κB/PI3K/Akt signaling pathway may result in anti-lymphangiogenesis molecular mechanisms in tumors.

Fucoidan is found in marine brown algae and is a polysaccharide that is rich in L-fucose and acidic sulfate groups, especially in the fucoidan extracted from *U. pinnatifida* sporophylls. In recent years, fucoidan has been used for the study of anti-tumor and anti-angiogenesis. According to our previous reports, fucoidan which extracted from *U. pinnatifida* sporophylls has anti-tumor effects: it suppressed the growth of tumors and induced apoptosis of cancer cells [11]. Additionally, fucoidan decreased the expression of VEGF-A, resulting in the inhibition of angiogenesis [12]. Furthermore, fucoidan suppressed the lymphatic metastasis of mouse hepatocarcinoma in normal and hypoxic environments [13, 14]. However, fucoidan direct effects of anti-lymphangiogenesis and anti-metastasis on LECs are barely.

We speculate fucoidan may play its anti-tumor and anti-metastasis activity by inhibiting lymphangiogenesis. Herein, we further evaluated the direct anti-lymphangiogenesis effect of fucoidan which extracted from *U. pinnatifida* sporophylls on human lymphatic endothelial cells (HLECs) *in vitro*. Moreover, we also established a mouse model to evaluate the inhibitory effect of fucoidan on the density of lymphatic vessels in tumor tissues. Our findings further revealed the potential suppressive mechanisms of fucoidan, which significantly inhibited the growth, migration and tubulogenesis of HLECs.

## RESULTS

### Fucoidan inhibits the proliferation of HLECs

**Figure 1:**
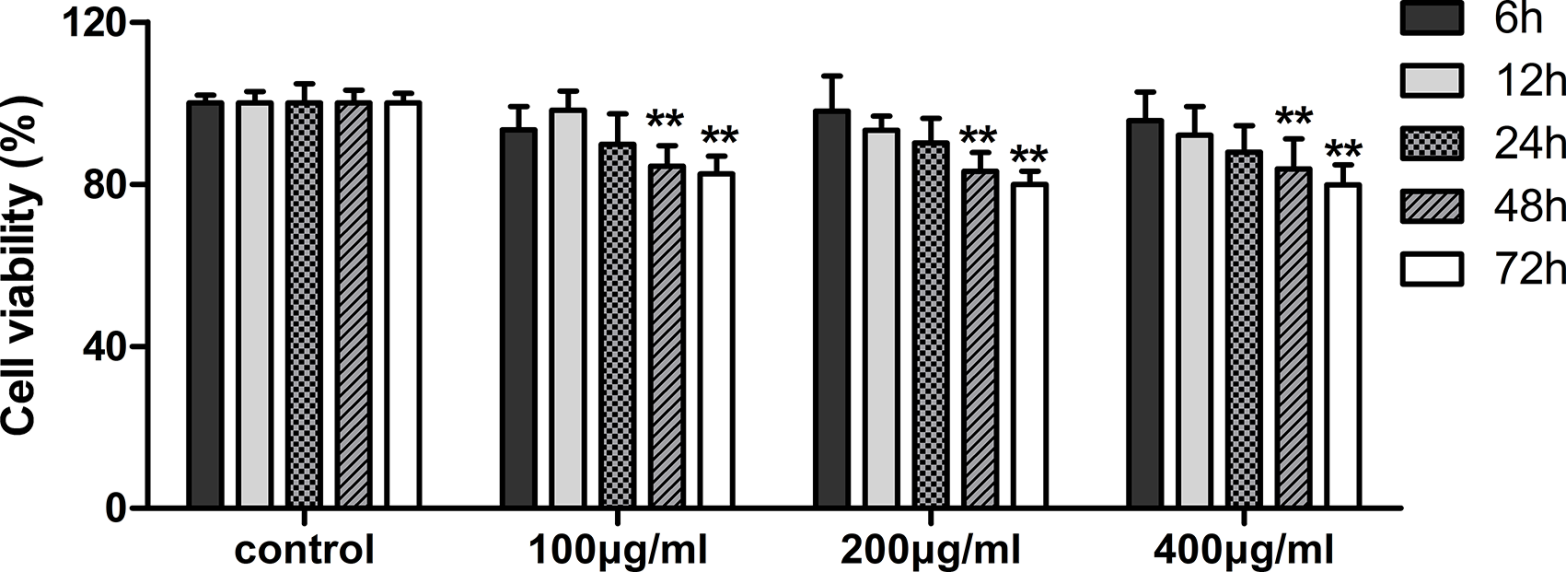
Effects of fucoidan on viability of HLECs Cell viability was detected in each group after 48 and 72 h in culture. Values are the mean ± SD from three independent experiments. **p < 0.01 versus the untreated control (two-way ANOVA).

**Figure 2:**
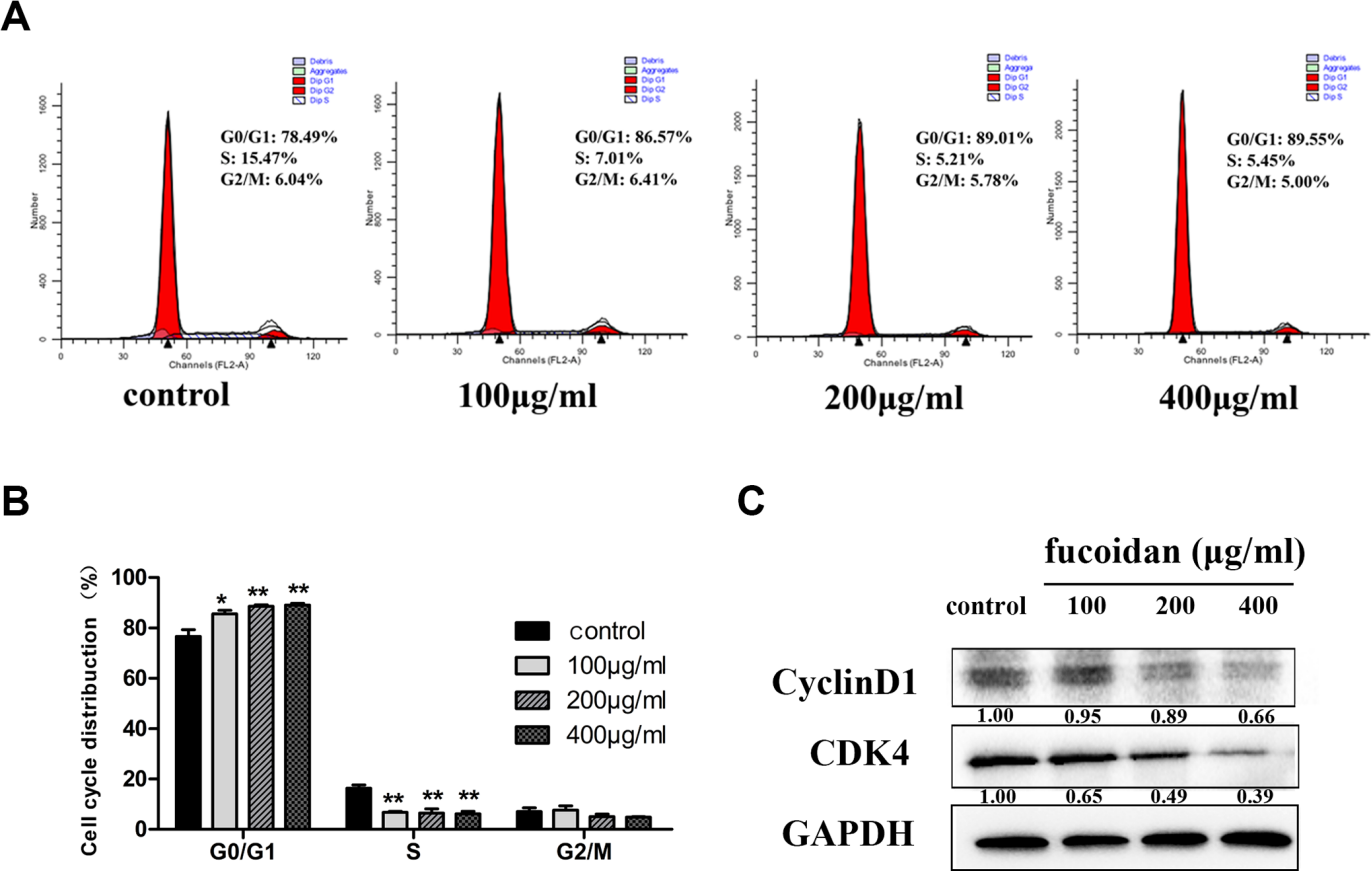
Effect of fucoidan on the cell cycle distribution in HLECs (**A**) Flow cytometry histograms and (**B**) cell cycle distributions for the G0/G1, S and G2/M phases assessed via flow cytometry, showing the G0/G1 phase was blocked by fucoidan after 48 h, inducing a decrease of cells in the S phase. (**C**) Effect of fucoidan on the protein expression levels of CDK4 and cyclin D1. Values are mean ± SD. *p < 0.05 and **p < 0.01 versus the untreated control (two-way ANOVA).

MTT assay was performed to assess cell viability of fucoidan (0, 100, 200 and 400 μg/ml) on HLECs over time (6, 12, 24, 48 and 72 h). Our data, as shown in Figure 1, demonstrated that fucoidan significantly decreased cell proliferation after treatment for 48 and 72 h. To establish the mechanism underlying the inhibition of proliferation, HLECs were treated with fucoidan (0, 100, 200 and 400 μg/ml) for 48 h and flow cytometry was performed to show that fucoidan induced an increase of cells in the G0/G1 phase, while cells in the S phase decreased (Figure 2A and 2B). In addition to this, the results showed that fucoidan decreased the expressions of CDK4 and cyclin D1 via western blotting, as shown in Figure 2C. These data indicated that fucoidan inhibited cell proliferation via the decreased expressions of CDK4 and cyclin D1, resulting in a block of the G1 phase of the cell cycle.

### Fucoidan inhibits the migration ability of HLECs

**Figure 3:**
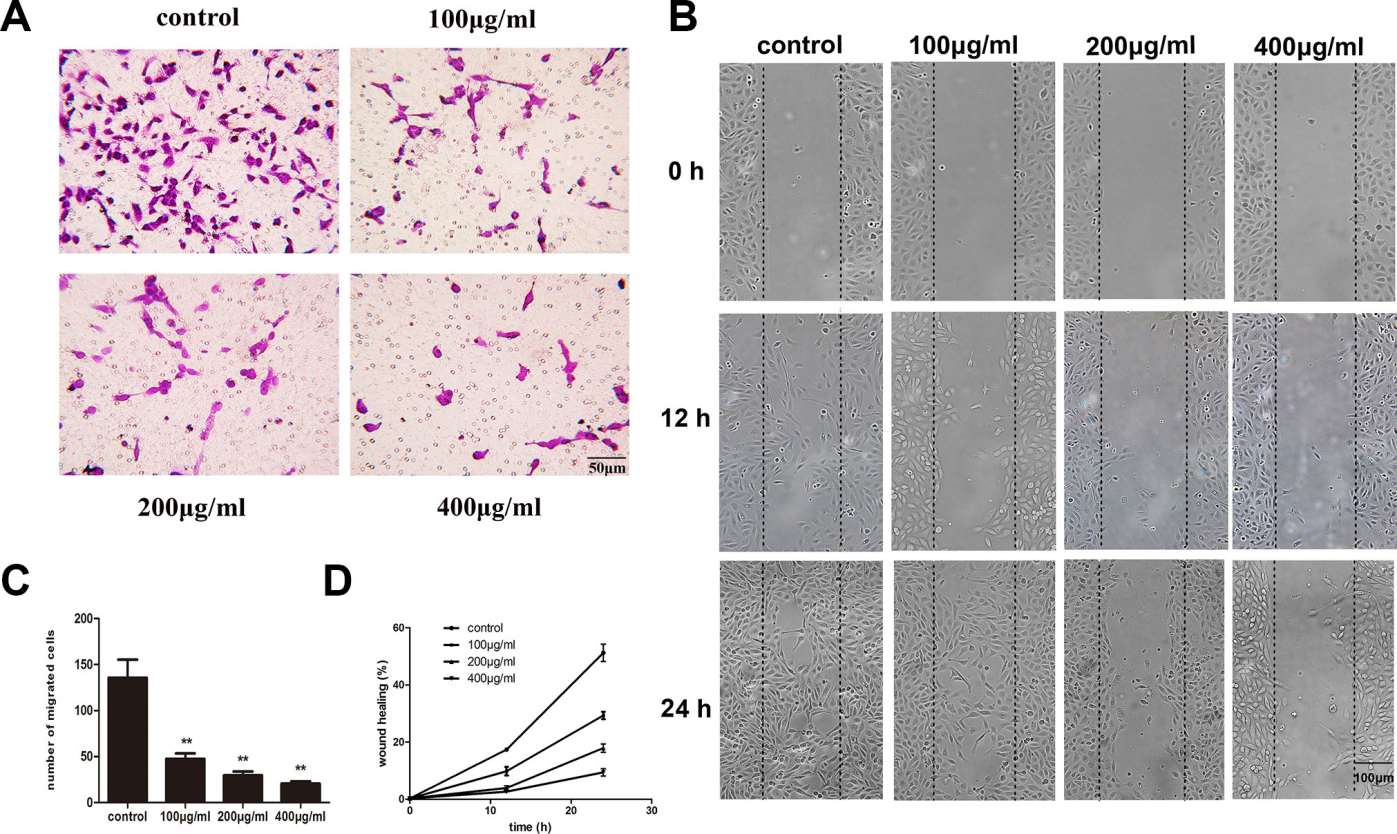
Fucoidan inhibits HLEC migration *in vitro* (**A**) Microscopy images (scale bar is 50 μm) and (**C**) number of migrated cells from the Transwell assay (migrated cells were stained and counted). The average value was calculated from five fields. Mean ± SD is shown; **p < 0.01 versus the untreated control (one-way ANOVA). (**B**) Quantification of wound healing areas was performed by using an ImageJ program. The graphs represent the means ± SD of triplicate experiments. (**D**) Wound scratch assays visually show the changes in cell migration with different concentrations of fucoidan over time (scale bar is 100 μm).

Lymphatic metastasis is a complex, multi-step process in malignant tumors and lymphangiogenesis is indispensable during this process and includes the migration of LECs. To explore whether fucoidan can inhibit HLEC migration *in vitro*, we used Transwell and wound scratch assays. Cells treated with fucoidan migrated significantly less (*p* < 0.01) compared with control cells in the Transwell assay (Figure 3A and 3C). The wound scratch assay visually represented the differences in the distances cells migrated after treatment with fucoidan (Figure 3B and 3D) and treated cells were seen to migrate less after 24 h compared to control cells.

### Inhibitory effect of fucoidan on the tube formation of HLECs

**Figure 4:**
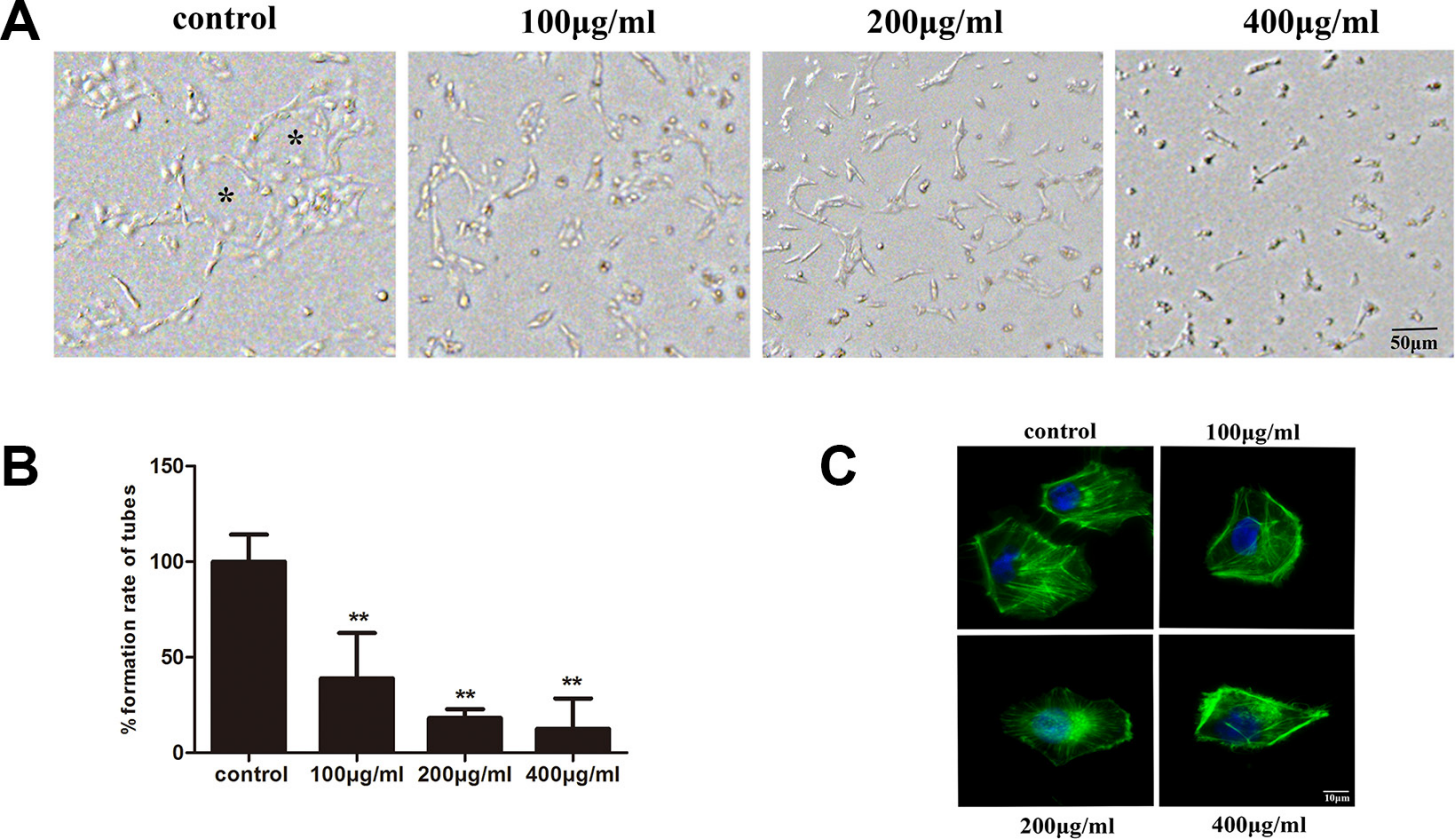
Fucoidan inhibits lymphatic tube formation in HLECs on matrigel and regulates cytoskeleton polarization of HLECs HLECs were incubated for 24 h in medium with or without fucoidan (0, 100, 200 and 400 μg/ml). (**A**) Photomicrographs of tube formation in HLECs (scale bar is 50 μm). The symbols (_*_) on the figure represents complete tubular structures. (**B**) Quantitative analysis of the rate from the number of tubes. Data represented as mean ± SD; **p < 0.01 versus the untreated control (one-way ANOVA). (**C**) Cells were incubated in medium with or without fucoidan (0, 100, 200 and 400 μg/ml) for 24 h. Cells were stained with phalloidin (green) and DAPI (blue), and observed (magnification, ×100).

To investigate the anti-lymphangiogenesis activity of fucoidan, the tube formation of HLECs *in vitro* was assessed. Control cells grown on matrigel for 24 h formed complete tubular structures, while fucoidan-treated HLECs did not form such structures. In addition, the number of tubes formed for the control group was much more than those treated with fucoidan (*p* < 0.01). Thus, treatment with fucoidan effectively decreased the tube formation of HLECs in a dose-dependent manner (Figure 4A and 4B).

The ability of both cell migration and tube formation is inseparably related with the cytoskeleton. The cytoskeleton supports cell shape, gives mechanical resistance to deformation and is also involved in cell movement by formation of pseudopodia. To explore whether fucoidan had an effect on microfilament distribution in HLECs, we stained cells with green-fluorescent phalloidin. The microfilament distribution was affected and cell polarity was changed in HLECs treated with fucoidan (Figure 4C). It is possible that fucoidan regulated the cytoskeleton rearrangement to inhibit cell motility.

### Fucoidan downregulates expression of VEGFR3 and PROX1 and activates NF-κB/PI3K/Akt signaling pathway

**Figure 5:**
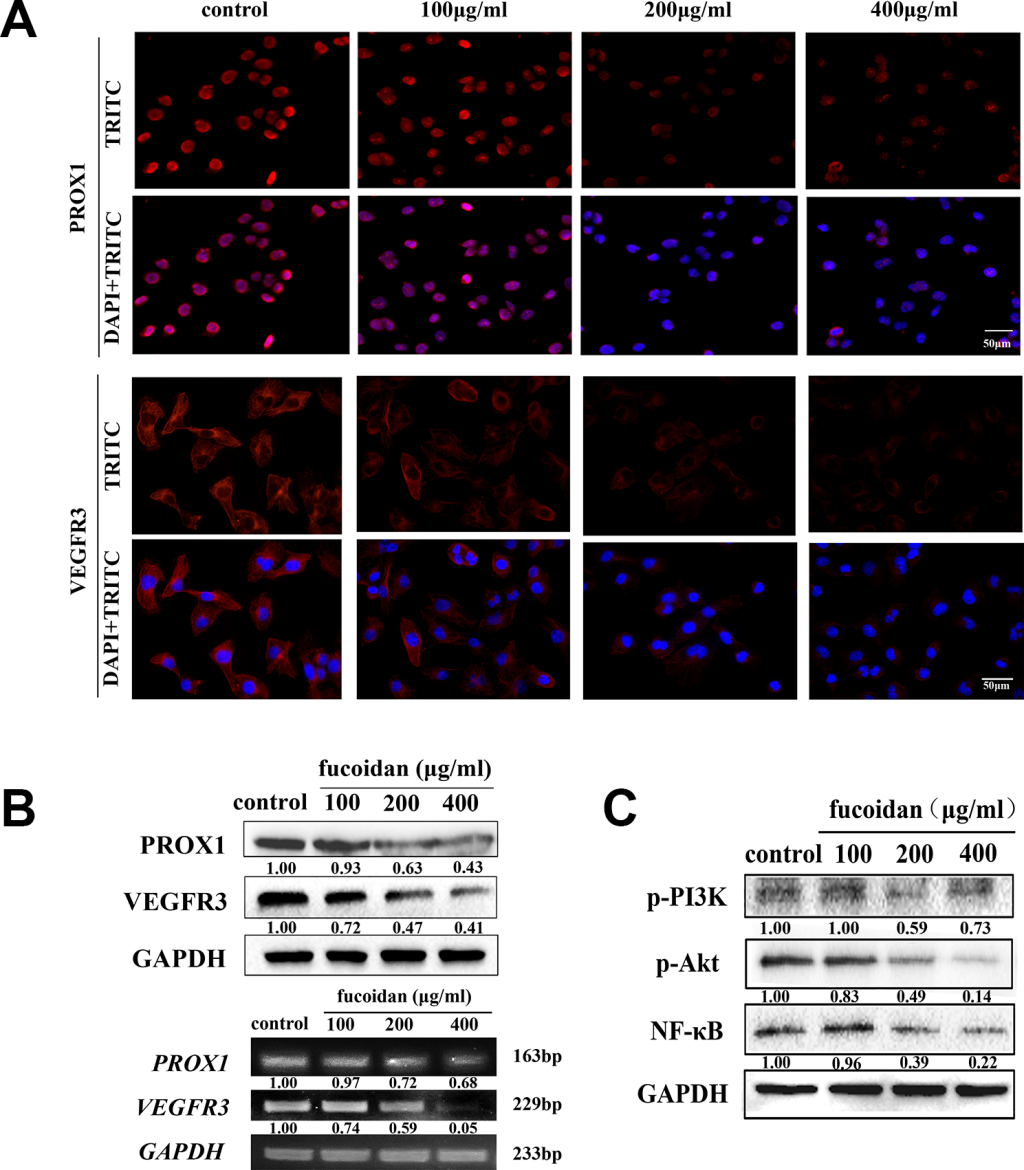
Fucoidan downregulates the expression levels of VEGFR-3 and PROX1 and VEGFR3-related signaling pathways (**A**) Microscopy images (scale bar is 50 μm) showing the immunofluorescence staining, which was performed with TRITC-labeled anti-VEGFR3 and anti-PROX1 (red) and DAPI (blue). (**B**) The protein expression levels of PROX1 and VEGFR3 were measured by western blot analysis; The transcription levels of *PROX1* and *VEGFR3* measured by RT-PCR. (**C**) Protein levels of p-PI3K, p-Akt and NF-κB in fucoidan-treated or control groups.

VEGFR3 and PROX1 are pivotal proteins present during the migration of LECs and lymphangiogenesis. To find out if fucoidan acts as an inhibitor of metastasis and lymphangiogenesis through reduction of the expressions of VEGFR3 and PROX1, immunofluorescence and western blotting were performed. The data demonstrated that the mRNA and protein levels of VEGFR3 and PROX1 were reduced with increasing concentrations of fucoidan, as shown in Figure 5A and 5B. Fucoidan at concentrations of 200 and 400 μg/ml notably decreased the expression of VEGFR3 (*p* < 0.05) and 200 and 400 μg/ml fucoidan significantly downregulated PROX1 expression (*p* < 0.05 and *p* < 0.01).

To examine whether fucoidan had an inhibitory effect on the activation of VEGFR3-induced signaling pathways, western blot analysis was carried out. The results indicated that the phosphorylation of PI3K and Akt and the protein level of NF-κB were all reduced by fucoidan in concentration-dependent manner (Figure 5C).

### Fucoidan inhibits tumor-induced lymphangiogenesis *in vitro*

**Figure 6:**
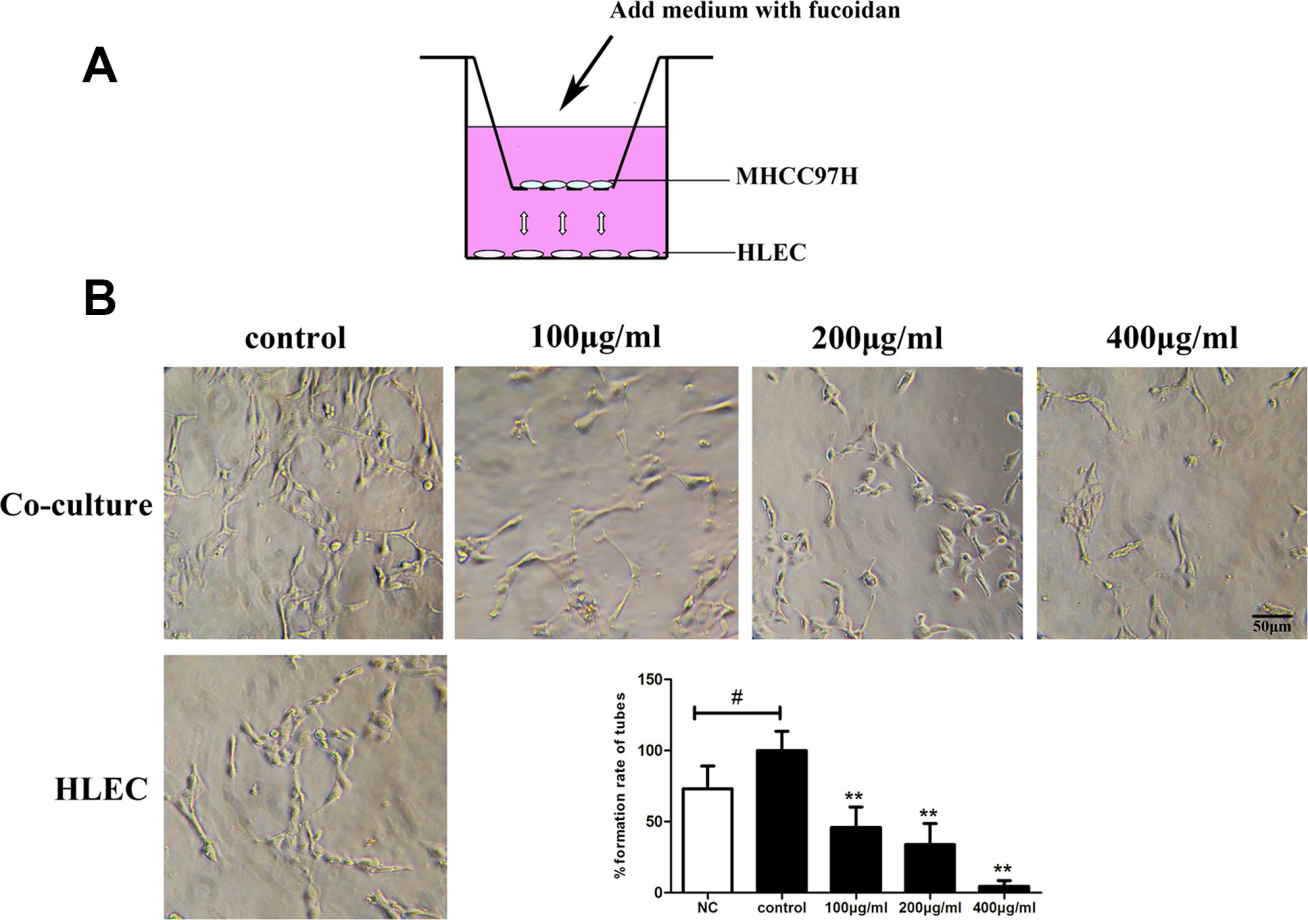
Fucoidan inhibits tumor-induced lymphangiogenesis *in vitro* (**A**) The ideograph of transwell co-culture system. MHCC97H cells and HLECs were cultured in insert wells and lower chambers, respectively. (**B**) Photomicrographs of tube formation in HLECs and the quantitative analysis shown on the right. NC stands for the negative control which HLECs cultured alone. Mean ± SD is shown; ^#^*p* < 0.05 versus the NC group and **p < 0.01 versus the co-culture control (one-way ANOVA).

To further analyze whether fucoidan had an inhibitory effect on tumor lymphangiogenesis, we assessed the ability of lymphatic tubular structures in co-culture system (Figure 6A). In the control group of co-culture, a larger number of tubular structures were formed and the lumens were connected closely. The results demonstrated that the ability of tubular structures is better in co-culture system than alone culture of HLECs (*p* < 0.05). In co-culture system, treatment with fucoidan effectively decreased the tube formation of HLECs in a dose-dependent manner (Figure 6B).

### Fucoidan inhibits lymphangiogenesis *in vivo*

**Figure 7:**
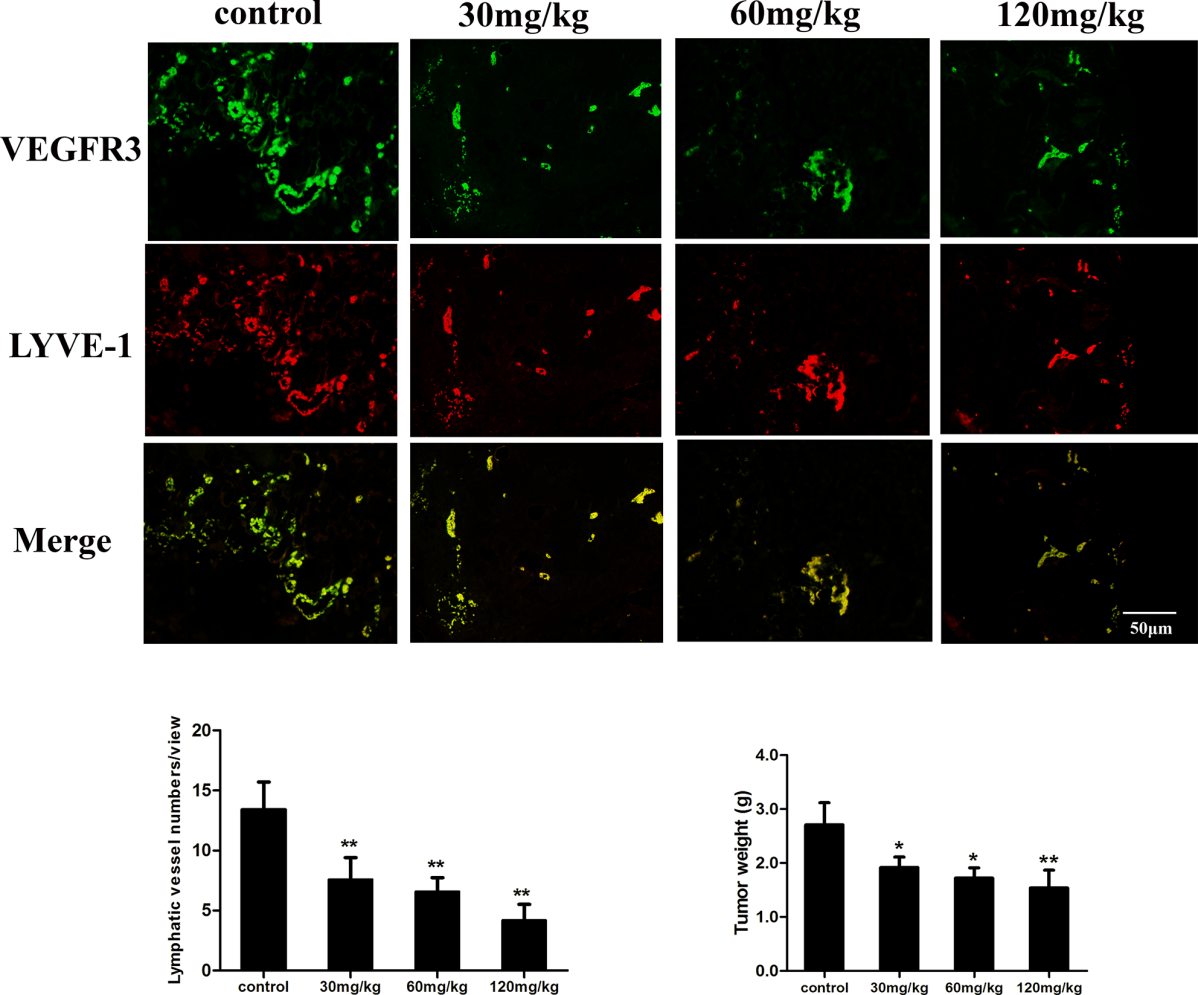
Fucoidan inhibits lymphangiogenesis *in vivo* Top: Immunofluorescence images of tumor lymphatic vessels from mice inoculated with Hca-F cancer cells. VEGFR3 antibody (green) and LYVE-1 antibody (red) were used in the assay. Bottom: Quantitative assessment of tumor LVD and tumor weight. Mean ± SD is shown; *p < 0.05 and **p < 0.01 versus the control.

We evaluated the anti-tumor and anti-lymphangiogenesis activity of fucoidan *in vivo*, whereby mice were inoculated with mouse hepatocellular carcinoma cells with high lymphatic metastatic activity (Hca-F cells). Immunofluorescent double staining was carried out on tumor tissues with VEGFR3 and LYVE-1, which are markers for LECs, and the results demonstrated that the mean micro-LVD of the tumors decreased from 13.4 ± 2.3 to 4.2 ± 1.3 as a result of treatment with fucoidan (120 mg/kg). As shown in Figure 7, the micro-LVD of mice treated with fucoidan was significantly reduced compared with the normal saline control group (*p* < 0.01). The average weight of tumor of fucoidan-treated mice were also significantly lower (*p* < 0.05 and *p* < 0.01) compared to those of the control. Thus, the analyses identified that fucoidan could inhibit tumor growth and lymphangiogenesis in tumor tissue *in vivo*.

## DISCUSSION

Metastasis of a malignant tumor is a critical characteristic of aggressive cancer and the cause of the majority of cancer deaths. Tumor metastasis via lymphatic vessels is a critical and early stage of metastatic dissemination of malignant tumors and is a prognostic factor of outcome [15]. Primary tumor cells invade existing lymphatic vessels and were transported to proximal lymph nodes accompanied with inducing lymphangiogenesis [16]. The formation of lymphatic vessels is considered as one fundamental step in tumor metastatic dissemination. Therefore, inhibition of lymphangiogenesis and blocking lymphatic metastasis are valuable for the development of novel therapeutic approaches to cancer.

Fucoidan is a natural, sulfated polysaccharide and recent studies have shown it to have anti-metastasis activity against various cancer types [17]. There have been several reports indicating that fucoidan and low molecular weight fucoidan present their anti-angiogenesis activity through the VEGF-regulated signaling pathway [12, 18]. However, the anti-lymphangiogenesis activity of fucoidan has, to date, not been investigated along with the direct effects on LECs.

LEC proliferation correlates with the formation of lymphatic network. Cyclin D1 and CDK4 are key positive regulators of transferring from G1 phase to S phase. Based on our data, the inhibitory role of fucoidan on HLEC proliferation has been validated. Most likely, this effect is linked to the retardation of the G0/G1 phase of the cell cycle by downregulation of cyclin D1 and CDK4 expression.

The ability of cell migration and tubulogenesis is inseparable from the roles of the microfilament distribution and cytoskeleton rearrangement [19]. In our study, fucoidan showed inhibitory effects on cell migration and tube formation *in vitro*. Thus, as expected, a change in the morphology of the HLEC cytoskeleton following treatment with fucoidan was noted, whereby fucoidan depressed the formation of pseudopodia.

By co-culture system, we can observe the interaction between two different cells to simulate environment of *in vivo*. We detected the effects of different concentrations of fucoidan on tube-like structure formation using transwell chamber noncontact co-culture of lymphatic endothelial cells and tumor cells. We verified that fucoidan inhibits tumor growth and tumor-induced lymphangiogenesis *in vitro*. In addition to the *in vitro* studies, lymphatic vascular density is one indicator to objective judge tumor lymphangiogenesis and it can predict the risk of lymphatic metastasis and tumor prognosis [20, 21]. Peritumoral lymphatic vessels around the tumor were diffuse distribution, lumen expanding, more conducive to tumor cell invasion. It is indicated that fucoidan had the inhibitory effects on lymphangiogenesis and significantly decreased the LVD of tumor tissues. So far, we have given evidence regarding the efficacious inhibitory effect of fucoidan on lymphangiogenesis *in vitro* and *in vivo*.

The homeobox transcription factor PROX1 is a master regulator and “switch” of the lymphatic system, and maintains the lymphatic vasculature during embryogenesis [22]. It is a useful, special LEC marker in normal tissues and is also a constitutive factor of LECs in diseased tissues and various cancers [23]. And VEGFR3 which is a valuable LEC marker, acts a remarkable role to facilitate lymphangiogenesis [24]. Here, we analyzed the expression of PROX1 and VEGFR3 following the treatment of HLECs with fucoidan versus untreated cells, and found that the expression levels of VEGFR3 and PROX1 were decreased with increasingly high concentrations of fucoidan. Therefore, our result suggested that fucoidan downregulates the expressions of VEGFR3 and PROX1 in HLECs consistent with its anti-lymphangiogenesis activity.

To investigate the inhibitory mechanisms of fucoidan, we further revealed that fucoidan stimulated the downstream signaling pathways of VEGFR3, including NF-κB, p-PI3K and p-Akt. The PI3K/Akt signaling pathway is involved in cell migration, adhesion, tumor angiogenesis and degradation of the extracellular matrix [25]. In view of these results, we suppose that fucoidan-related inhibition mechanisms of lymphangiogenesis occur via inhibition of NF-κB/PI3K/Akt signaling pathways.

In conclusion, we demonstrated that fucoidan suppresses the progression of lymphangiogenesis *in vitro* and exerts anti-tumor and anti-lymphangiogenesis in tumor model *in vivo*. Fucoidan induced the inhibition of cell growth via G1/G0 phase cell cycle arrest and had anti-migration activity, as well as an inhibitory effect on tube formation. The inhibitory role of fucoidan on HLECs was likely due to its ability to decrease the expression level of PROX1 and downregulate VEGFR3 activation, which may be associated with the interference of PI3K/Akt signaling in LECs. Thus, fucoidan could be used as a promising anti-tumor agent in a novel therapeutic approach within a clinical setting.

## MATERIALS AND METHODS

### Reagents and antibodies

The sources and characteristics of the fucoidan used in this study have been previously reported [13]. Fucoidan was diluted in Roswell Park Memorial Institute 1640 (RPMI 1640) complete medium at a concentration of 800 μg/ml. RPMI 1640 medium and fetal bovine serum (FBS) were purchased from Gibco (Life Technologies, South America). Trypsin was obtained from Hyclone (Thermo Fisher Scientific, Carlsbad, CA, USA).

The following primary antibodies were used in this study: VEGFR3, lymphatic vessel endothelial hyaluronan receptor 1 (LYVE-1) and phospho-Akt (p-Akt; Santa Cruz, CA, USA); glyceraldehyde 3-phosphate dehydrogenase (GAPDH) and cyclin-dependent kinase 4 (CDK4; Sangon Biotech, Shanghai, China); PROX1 and cyclin D1 (Boster, Wuhan, China); NF-κB (Zhongshan Biotech, Beijing, China) and p-PI3K (ImmunoWay, Newark, DE, USA).

### Cell lines and cultures

HLECs, obtained from Sciencell Research Laboratories (Carlsbad, CA, USA; http://sciencellonline.com/), were cultured in RPMI 1640 medium with 15% fetal bovine serum (FBS) and kept at 37°C in a humidified chamber containing 5% CO_2_ and 95% air. The human hepatocarcinoma cell line MHCC97H, obtained from the KeyGEN biotech (Jiangsu, China) and cultured in DMEM containing 10% FBS. The mouse hepatocarcinoma cell Hca-F, established and stored by Department of Pathology, Dalian Medical University [26]. Cells from the 3rd to 8th passage were used for all experiments.

### Assessment of viability and cell proliferation

A 3-(4,5-dimethylthiazol-2-yl)-2,5-diphenyltetrazolium bromide (MTT; Sigma, MO, USA) assay was used to analyze the effects of fucoidan on HLEC viability *in vitro*. HLECs (1.0 × 10^4^ cells/well) were cultured in 96-well culture plates for 4 h and then treated with different concentrations (0, 100, 200 or 400 μg/ml) of fucoidan for 6–48 h. MTT (5 mg/ml) was added to the cells and the plate was incubated for an additional 4 h. Dimethyl sulfoxide (DMSO) was added to dissolve the formazan crystals and the absorbance (*A*) was measured with a microplate spectrophotometer (Thermo Fisher Scientific, Carlsbad, USA). The cell viability was calculated according to the following equation.

Cell viability (%) = (*A*_treated_ − *A*_blank_)/(*A*_control_ − *A*_blank_) × 100%.

Separate experiments were performed in triplicate.

### Flow cytometry

HLECs (1 × 10^6^ cells/ml) were incubated for 48 h at 37°C and then suspended in 500 μl 75% ice-cold ethanol for over 2 h at 4°C. The resulting cell pellet was collected via centrifugation of 800 rpm for 5 min and RNase A (100 μl) was added prior to incubation for 30 min at 37°C. Propidium iodide (400 μl) was used as a DNA stain. The cell cycles of the experimental and control groups were analyzed using a FACSCalibur™ flow cytometer (BD, USA).

### Migration assay *in vitro*

The anti-migration effect of fucoidan on HLECs was assessed using a Transwell migration assay and a wound scratch assay. Transwell was adopted to assay the chemotactic motility of HLECs. Cells (5 × 10^4^ cells/well) were plated on the upper Transwell chamber (6.5 mm diameter inserts; 8.0 μm pore size) and treated with different concentrations of fucoidan (0–400 μg/ml) in serum-free medium, while the inside lower chamber contained fresh medium in the absence of fucoidan. After 24 h in culture, cells were fixed with 70% ethanol and stained with 10% crystal violet in order to count the migrated cells at the undersurface of the filter. At least five visual fields were counted for each group to obtain an average.

The migratory activity of HLECs was also measured using a wound scratch assay. Briefly, cells (80–90% confluency) were conformably wounded using a micropipette tip and washed with phosphate-buffered saline (PBS) to remove the suspended cells. The cells were then incubated in fresh medium diluted with 100, 200 or 400 μg/ml fucoidan. The images were collected on an inverted microscope.

### Tube formation assay *in vitro*

A matrigel-based tube formation assay was performed to analyze the effect of fucoidan on the formation of lymphatic-like structures *in vitro*. Briefly, HLECs (1 × 10^4^ cells/well) were plated in 96-well plates that were pre-incubated with matrigel (Sigma, MO, USA) at 37°C for 24 h. An inverse microscope was used to evaluate and image at least three random fields per well in triplicate [12]. The formation rate of tubes was calculated according to the following equation.

Formation rate (%) = tube quantity_treated_/tube quantity_control_ × 100%.

### Western blotting

To examine the expression levels of proteins in HLECs under the influence of fucoidan, cells were first treated with fucoidan for 24 h and then lysed in radioimmunoprecipitation assay (RIPA) lysis buffer (Thermo scientific, USA). Equal amounts of protein (40 μg) were measured using the Bradford method and resolved by 12% sodium dodecyl sulfate polyacrylamide gel electrophoresis (SDS-PAGE) and electro-transferred onto nitrocellulose (NC) membranes. Membranes were incubated overnight at 4°C with relevant primary and peroxidase-conjugated secondary antibodies; finally, the bands were detected with an ECL system (Bio-Rad Laboratories, CA, USA).

### Reverse transcription polymerase chain reaction (RT-PCR) analysis

Cells treated with fucoidan were cultured and, after 24 h, total RNA was extracted using TRIzol reagent (Invitrogen, Carlsbad, CA, USA). Using a two-step RT-PCR kit (Invitrogen, Carlsbad, CA, USA), RNA was reverse-transcribed to cDNA. For standardization, *GAPDH* was used as an internal control. The following primers: *GA* PDH, 5′-TGGCACCCA GCACAATGAA-3′ (sense) and 5′-CTAAGTCATAGTC CGCCTAGAAGCA-3′(antisense); *PROX1*, 5′-ACAAGC TCAGTGCCGTTTGA-3′ (sense) and 5′-CTAGGAGC TGCACGTTAGGC-3′(antisense); *VEGFR3*, 5′-ATTCCC CATGACCCCAACGA-3′(sense) and 5′-GTAAAACACC TGGCCTCCTCG-3′ (antisense). The PCR products were separated via electrophoresis on 1% agarose gel and observed by ethidium bromide staining using ultraviolet (UV) transillumination.

### Immunofluorescence and cytoskeleton staining assay

HLECs were fixed and perforated by 0.2% Triton X-100. After washing with PBS three times for 5 min, cells were blocked with 1% bovine serum albumin (BSA) for 1 h and incubated at 4°C with rabbit polyclonal anti-PROX1 and anti-VEGFR3 antibodies. Using appropriate tetramethylrhodamine-(TRITC-) conjugated secondary antibodies, cells were incubated at 37°C for 1 h and then counterstained with 4′,6-diamidino-2-phenylindole (DAPI) to reveal cell nuclei.

For cytoskeleton staining, cells were fixed in 3.7% methyl aldehyde for 10 min, washed with PBS containing 0.1% TritonX-100 for 5 min and stained for 20 min at room temperature with green-fluorescent phalloidin (KeyGEN Biotech, Nanjing, China). After washing three times, cells were examined under a fluorescent microscope (Leica, Solms, Germany).

### Co-culture and tube formation assay

In this assay, co-culture system was established with HLEC and MHCC97H to evaluate the ability of tumor induced lymphangiogenesis using 24-well plates. In transwell co-culture system which includes insert wells (6.5 mm diameter inserts; 0.4 μm pore size) and plates, MHCC97H (1 × 10^5^ cells/well) were plated on the upper chamber and HLECs (5 × 10^4^ cells/well) were plated in 24-well plates which were pre-incubated with matrigel. An inverse microscope was used to evaluate and image at least three random fields per well in triplicate.

### Lymphangiogenesis assay *in vivo*

Male 615 mice, 6–8 weeks old, were purchased from Dalian Medical University Experimental Animal Center. The tumor model was established with Hca-F cells (5 × 10^6^ cells, 40 μl), which were inoculated subcutaneously into the left armpit of twenty-four mouse subjects. After 48 h, mice were then randomly divided into control and experimental groups, with normal saline (NS) as a control, and fucoidan (30, 60, 120 mg/kg) or NS were intragastrically administered to mice everyday. Mice were euthanized on day 14, the tumors *in situ* were isolated and fixed in 10% neutral formaldehyde, paraffin embedded, sectioned and the lymphatic vessel density (LVD) of the tumors was quantified by immunofluorescence stain. Animal studies were performed following the appropriate ethical criteria and approved by the Committee for the Care and Use of Animal Subjects at the Dalian Medical University.

### Statistical analysis

Data were expressed as the mean ± standard deviation (SD). Significant differences were evaluated by one-way or two-way analysis of variance using Prism 5 (Version 5.04, Graphpad Software, Inc., La Jolla, CA, USA) [27]. Statistical significance was defined as *p* < 0.05 and *p* < 0.01. All experiments were performed at least three times for quantitative comparison.

## Abbreviations

HLEC: human lymphatic endothelial cell
PROX1: prospero homeobox 1
VEGFR3: vascular endothelial growth factor receptor 3
LYVE-1: lymphatic vessel endothelial hyaluronan receptor 1
PI3K: phosphoinositide 3-kinase
Akt: serine/threonine kinase
LVD: lymphatic vascular density
